# The Surgical Uses of Anti-Streptococcic Serum

**Published:** 1897-04-10

**Authors:** 


### THE SURGICAL USES OF ANTI-STREPTOCOCCIC SERUM.

This month's *Practitioner* contains a paper by Mr. Watson Cheyne on the uses of anti-streptococcic serum. He thinks that in the cases which have been published, in which this substance has been used in cases of streptococcal infection, the proof that the recovery has been due to the injections is not altogether satisfactory. It is clear, however, that the serum is an active substance, and does have a considerable influence over the local process, and he thinks that the experimental evidence is strongly in favour of the view that the serum ought to exercise a prophylactic action. Now there are certain operations, such as those about the pharynx, the tonsil, and the base of the tongue, after which septic pneumonia is a frequent cause of death. This form of pneumonia is due to infection by the streptococcus pyogenes, and if the serum will act as a prophylactic against this the great bugbear of the operation will be removed. Two cases are related in which serious operations were performed for the removal of malignant growths extensively involving the parts about the lower jaw and the pharynx, exactly the sort of cases in which sloughing of the surface of the wound, offensive odour, inflammation, and septic pneumonia are very likely to occur. In the one case, two days before the operation, he injected 20 c.cm. of antistreptococcic serum, and on the day before the operation 10 c.cm., which dose was repeated again the day after. In the other case 20 c.cm. were injected one day, 20 the next, and 10 the next, the day of the operation. The course of both these cases was markedly different from what usually occurs after similar operations; no sloughing of the surface of the wound, no odour of the breath, no inflammation, and no tendency whatever to any septic infection or any septic pneumonia. In both cases the wound healed without any trouble whatever. In a third case 90 c.cm. of the serum were injected during the four days preceding the operation, and here again there was the same freedom from septic trouble and absence of smell, but unfortunately the patient died suddenly on the fifth day from cerebral embolism. Mr. Watson Cheyne says that if one can, by means of the serum, prevent the occurrence of septic pneumonia and those other diffuse septic processes which are so apt to occur in these cases, a great step in advance will have been made in regard to the feasibility of such operations.

